# A Chiral Metal-Organic 1D-Coordination Polymer Upon Complexation of Phenylene-Bridged Bipyrrole and Palladium (II) Ion

**DOI:** 10.3389/fchem.2020.613932

**Published:** 2020-12-01

**Authors:** Kumiko Nishinaka, Jiandong Han, Dongli Han, Yue Liu, Yanqing Du, Meiling Wang, Chaolu Eerdun, Nobuyasu Naruse, Yutaka Mera, Yoshio Furusho, Akihiko Tsuda

**Affiliations:** ^1^Department of Chemistry, Graduate School of Science, Kobe University, Kobe, Japan; ^2^Department of Pharmaceutical Sciences, Inner Mongolia Medical University, Hohhot, China; ^3^Department of Chemistry, Shiga University of Medical Science, Otsu, Japan

**Keywords:** coordination polymer, helical polymer, complex, π-conjugation, chirality

## Abstract

Metal-organic 1D-coordination polymers, having unique electronic and optical properties, are expected to be a novel advanced functional material capable of fabricating smart plastics, films, and fibers. In this study, we have synthesized a novel metal-organic 1D-coordination polymer composed of a phenylene-bridged bipyrrole bearing *N*-alkylimino groups (BPI) and palladium(II) ion. The BPI and Pd(II) form square planar bis(bidentate) complex to form a metal coordinated π-conjugation polymer (Poly-BPI/Pd). It is stable in solutions at room temperature, and allowed measurement of its average molecular weight in SEC (*M*_w_ = 106,000 and *M*_n_ = 18,000, *M*_w_/*M*_n_ = 5.88). It also provided a reversible multi redox profile in cyclic voltammetry, most likely originating from strong π-electronic interactions between the BPI components via Pd ion. A variety of substituent groups can be attached to the imino-nitrogens of BPI. A coordination polymer composed of a BPI derivative bearing chiral alkyl chains and Pd(II) showed strong circular dichroism (CD) in the solution due to the unidirectional chiral conformation of the BPI components in the polymer backbone.

## Introduction

Coordination polymers (CPs) having two- and three-dimensional network structures such as nanosheets, metal-organic frameworks (MOFs), and porous coordination polymers (PCPs) have been actively studied (Wang et al., 2017; Sahadevan et al., 2018; Gu et al., 2019). These materials have attracted intense interests in terms of potential wide applications in catalysis, magnetism, luminescence, electrical conduction, chemical sensing, and especially in gas storage and separation (Sahay et al., 2014; Stavila et al., 2014; Kirchon et al., 2018). On the other hand, less attention has been paid to one-dimensional coordination polymer (1D-CP), which is expected to have characteristic molecular functions originating from its repetitive metal-organic components and softness of the linear polymers (Noro et al., 2009; Liu et al., 2017). In the reported examples, the 1D-CPs appear mainly in the solid-state, but readily dissociate into the constitutive ligands and metals and/or aggregate randomly in solution. For this reason, it is a challenging subject to isolate and functionalize the metal-organic 1D-CP in the solution. As a limited example, Nishihara and coworkers reported 1D-CPs comprising bridging dipyrrin ligands and divalent metal ions (Zn^2+^, Ni^2+^, and Cu^2+^) (Matsuoka et al., 2015). Their 1D-CPs are stable in solution but include a non-conjugated chromophoric ligand that allows weak electronic communications over the polymer backbone. In the present study, with an expectation to increase the electronic interactions between the components, which provides unique electronic and optical properties, we have designed a novel metal-organic 1D-CP with a π-conjugated ligand and metal ion.

**Figure 1:**
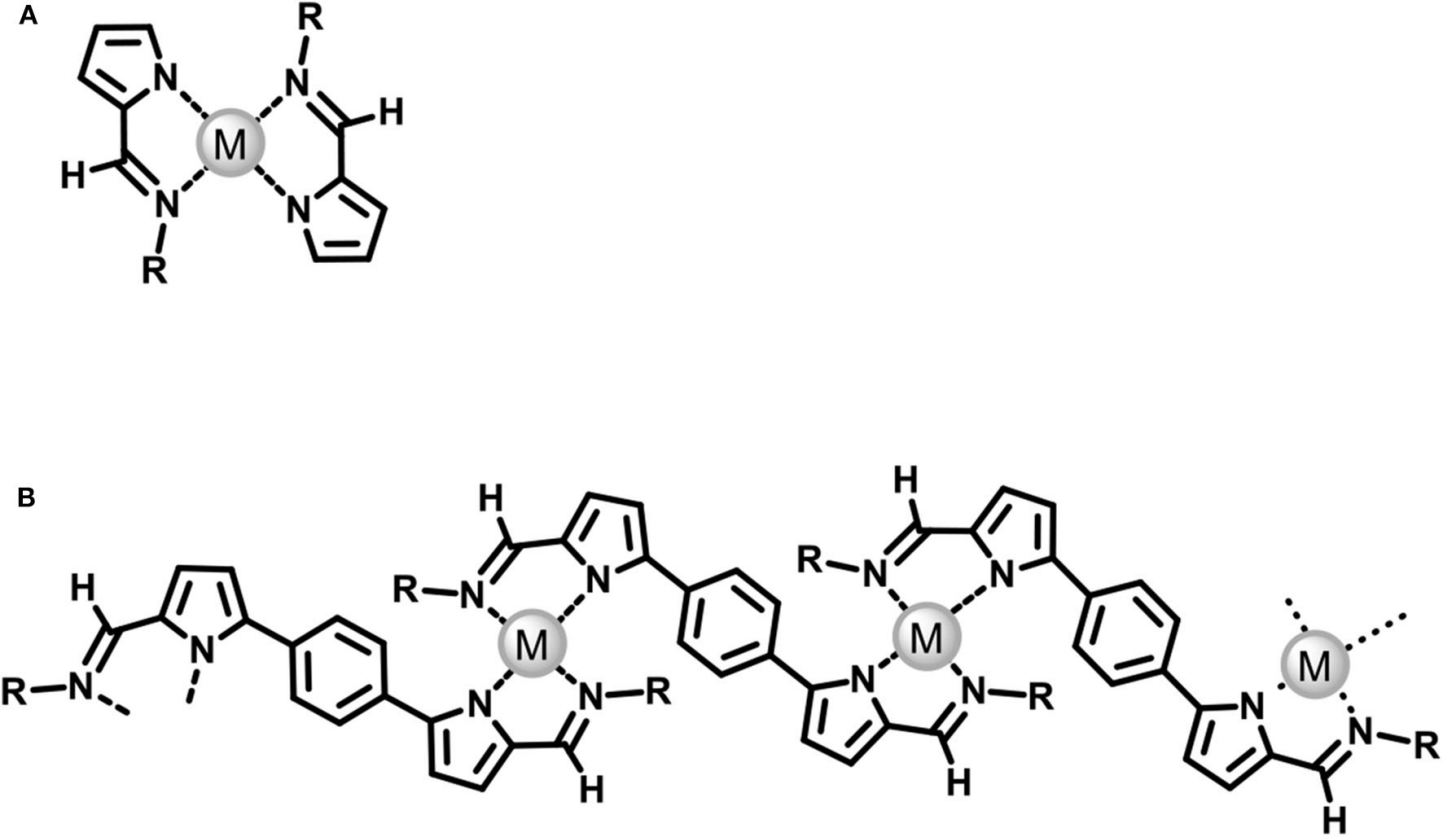
**(A)** 2:1 complex of *N*-substituted pyrrole-2-aldimines and transition metal ion reported previously, **(B)** 1:1 complex of bis(*N*-substituted pyrrole-2-aldimines) and transition metal ion studied in this study.

We previously reported the synthesis of a π-conjugated phenylene-bridged bipyrrole bearing *N*-alkylimino groups (**BPI-1**), and found that it is an acid-responsive single trichromatic luminescent dye capable of emitting pure white light (WL) (Imamura et al., 2017). The blue-light-emitting **BPI-1** exhibits dramatic color changes in fluorescence to orange and green upon mono- and diprotonation, respectively, providing a wide emission band in the range of λ = 400–800 nm that provide WL when the compound is in a dynamic equilibrium between the three states. With this **BPI-1** and its derivatives as a π-conjugated organic component (bis-bidentate ligand), we have newly designed and synthesized metal-organic 1D-CPs upon complexation with palladium(II) ion (Poly-**BPI**/**Pd**). *N*-Substituted pyrrole-2-aldimines are known to form metal complexes with transition metal ions (Figure 1A) (Holm et al., 1966; Yeh and Barker, 1967). We expected that the **BPI** monomers are directly connected with Pd(II) ions to form square-planar bis(bidentate) complexes to form a metal coordinated π-conjugation polymer (Figure 1B). We found, herein, that Poly-**BPI**/**Pd** is stable in the solutions at room temperature and shows a reversible multi redox behavior. Further, its derivative having chiral alkyl chains shows strong circular dichroism (CD) in the solution due to the unidirectional chiral conformation of the **BPI** components in the polymer backbone.

## Results and Discussion

### Synthesis and Characterization of Metal–Organic Coordination Polymers

**Scheme 1:**
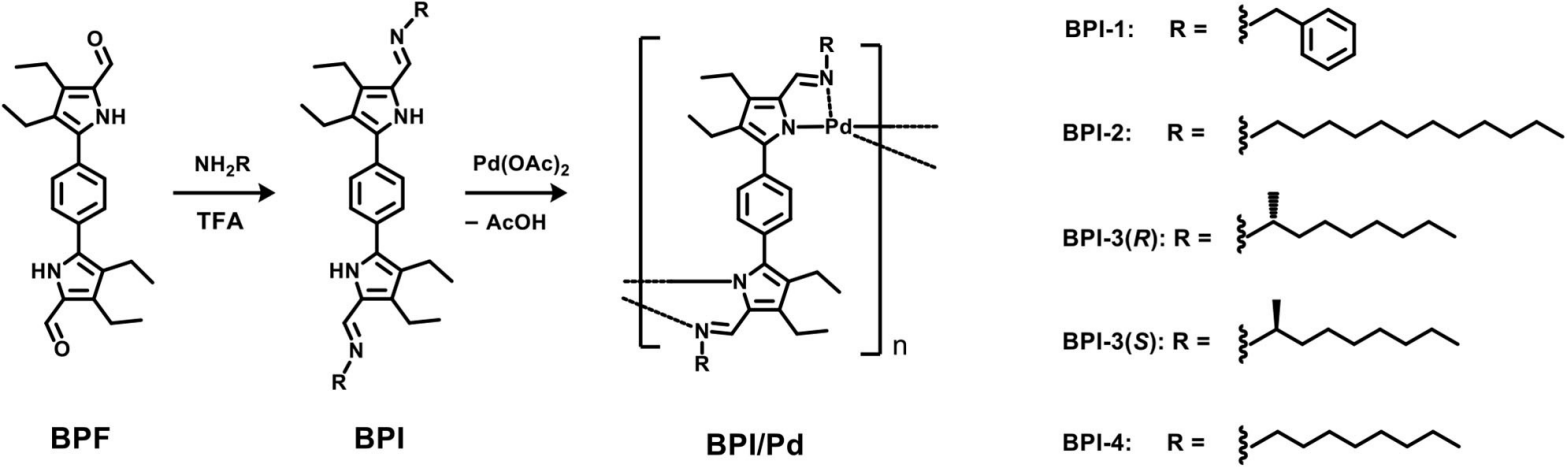
Synthesis of a series of **BPI** and **BPI**/**Pd** polymers.

We synthesized imino-substituted bipyrrole derivatives **BPI-1**–**4**, containing a 1,4-phenylene spacer according to the previously reported procedures Scheme 1 (Setsune et al., 2015). **BPF** bearing formyl groups at pyrrolic α-positions were synthesized in advance. Then, the formyl groups were converted to the corresponding imines bearing various functional groups on the nitrogen atom through condensation reactions with the amine in the presence or absence of an acid catalyst (isolated yields 75–96 %).

**Figure 2:**
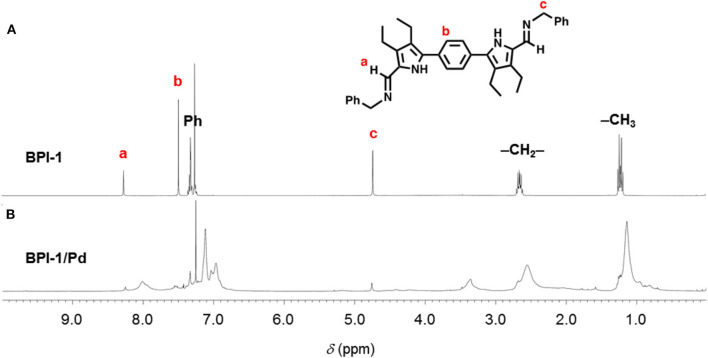
^1^H NMR spectra (400 MHz, CDCl_3_) of **(A) BPI-1** and **(B)** a product formed upon mixing **BPI-1** and Pd(OAc)_2_ at 1:1 ratio.

We initially attempted the synthesis of 1D-CPs with **BPI-1**, bearing *N*-benzyl groups, and transition metal salts such as Zn(OAc)_2_, Cu(OAc)_2_, and Pd(OAc)_2_. **BPI-1** and metal salt were mixed in a 1:1 ratio in a mixture solution of CH_2_Cl_2_/CH_3_OH (5:1) and stirred overnight at room temperature. In these experiments, only the sample solution containing **BPI-1** and Pd(OAc)_2_ provided a precipitate, which is slightly soluble into organic solvents such as CHCl_3_ and CH_2_Cl_2_, likely due to the complexation. ^1^H NMR spectrum of **BPI-1** in CDCl_3_ provides characteristic singlet signals corresponding to the protons on imine, phenylene, and benzyl groups at δ = 8.27, 7.49, and 4.73 ppm, respectively (Figure 2). These peaks become broad and are shifted to the higher magnetic field region at 8.01, 7.33–6.97, and 3.36 ppm, respectively, upon complexation with Pd(II) ion. Similar changes were observed for other peaks corresponding to the phenyl and ethyl exteriors. These ^1^H NMR spectral features indicate the formation of the certain polymers (Berl et al., 2002).

**Figure 3:**
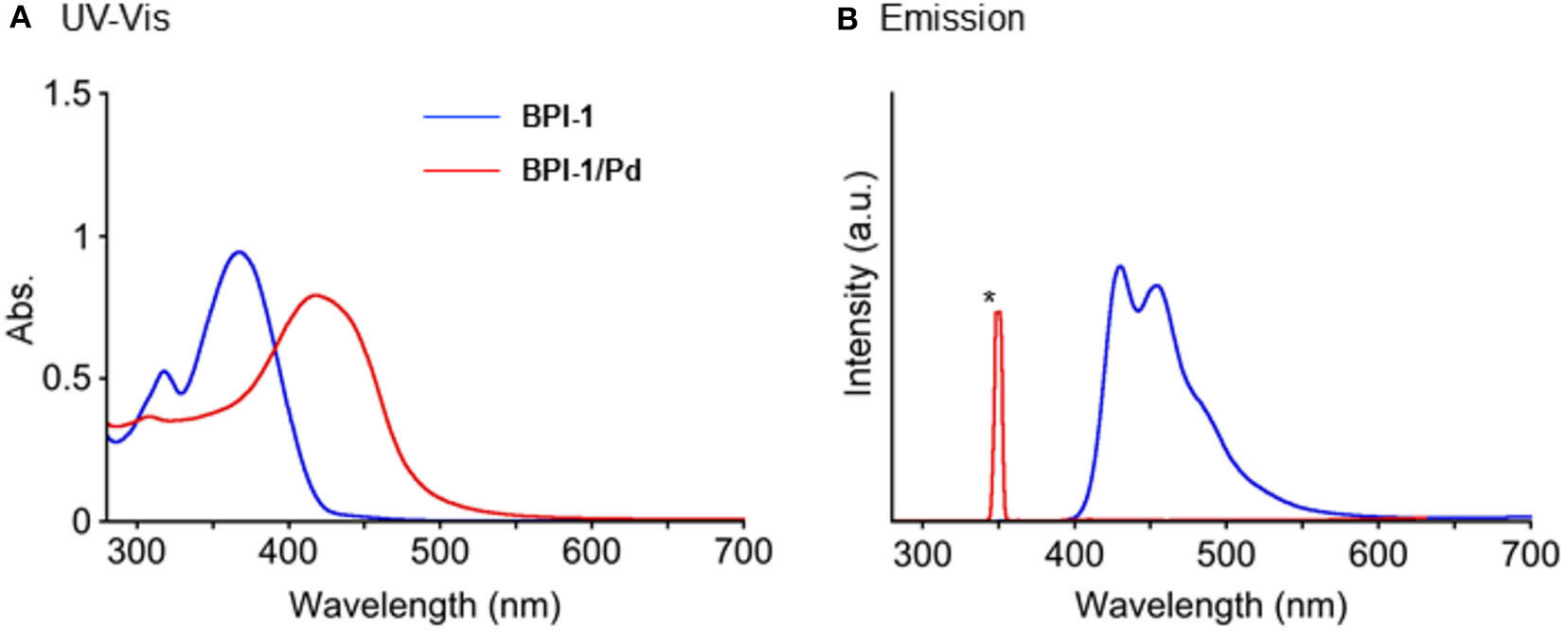
**(A)** UV-Vis absorption spectra and **(B)** fluorescence spectra upon excitation at 350 nm of **BPI-1** and **BPI-1**/**Pd** complex in CH_2_Cl_2_ at 25°C. [**BPI-1**] = 2.28 × 10^−5^ M, [**BPI-1**/**Pd** complex] = 1.92 × 10^−5^ M. *Stray light.

On the other hand, in UV-Vis absorption spectroscopy, **BPI-1** showed a large red-shift of its lowest energy absorption band from λ_max_ = 368 nm to 419 nm in CH_2_Cl_2_ upon complexation with Pd(II) (Figure 3), which may mainly originate from exciton coupling of **BPI-1** array, owing to the electric transition dipole for the long-axis polarized π–π^*^ transition (*vide infra*) (Ahmad et al., 2009; Li et al., 2019). Further, the emission spectrum of **BPI-1**, observed at 400–600 nm with λ_max_ = 426 and 450 nm, disappeared upon the complexation. This observed quenching behavior may be ascribed to the heavy atom effect due to Pd ion (Drzewiecka-Matuszek et al., 2005). These observed spectral features strongly indicate the formation of a metal-organic CP. However, its low solubility into organic solvents does not allow measurement of the average molecular weight in SEC.

**Figure 4:**
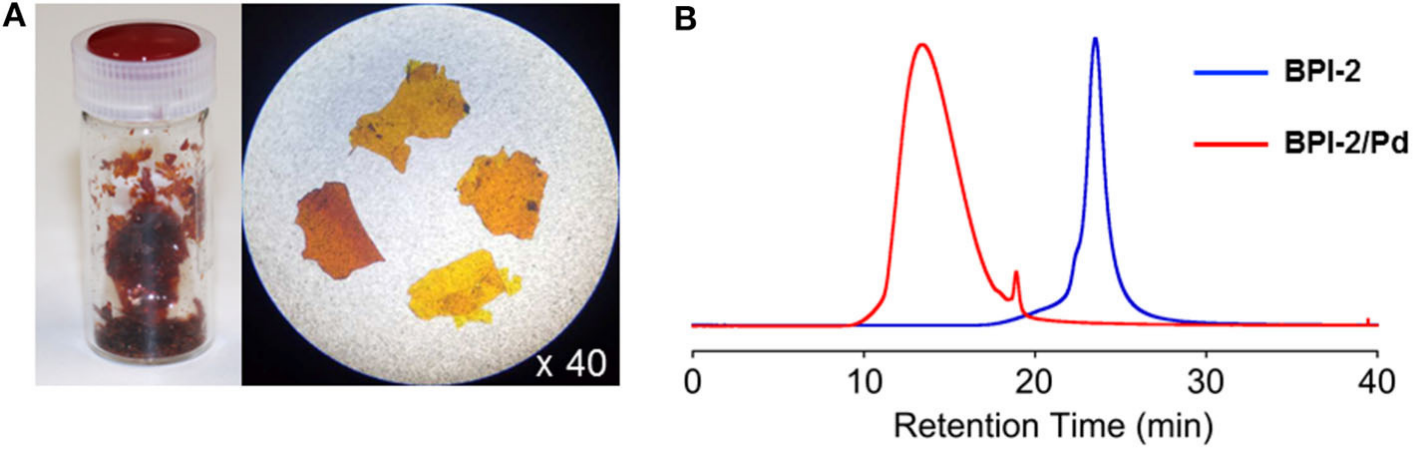
**(A)** Photos of film-like solid samples of **BPI-2/Pd** complex. **(B)** SEC profiles of **BPI-2** and **BPI-2/Pd** complex monitored at 424 and 420 nm, respectively, in SEC. [Conditions] Column: TOSOH TSKgel G4000H_XL_ + G4000H_HR_, Solvent: CHCl_3_, Flow speed: 1 mL/min, Temperature: 20°C.

We then synthesized **BPI-2**, having *N*-dodecyl chains, to increase the solubility of the product into organic solvents. **BPI-2** was mixed with 2 equiv. amounts of Pd(OAc)_2_ in CH_2_Cl_2_, and stirred for 2 days at room temperature to give the complex soluble into organic solvents such as CHCl_3_ and CH_2_Cl_2_. The **BPI-2**/**Pd** complex was thus isolated through extraction with CH_2_Cl_2_ and water to give a film-like solid after evaporation of the solvent (Figure 4A). Spectral changes of **BPI-2** in ^1^H NMR, UV-Vis absorption, and fluorescence spectroscopies upon complexation with Pd(II) were almost the same as those observed in **BPI-1** as described above (Supplementary Figures 1, 2). **BPI-2**/**Pd** is stable in solutions and was subjected to the SEC measurements to estimate average molecular weight. SEC was conducted with CHCl_3_ as an eluent, and it revealed that **BPI-2**/**Pd** complex has shorter retention time (13.4 min) than **BPI-2** monomer (23.5 min) (Figure 4B). The average molecular weight of **BPI-2**/**Pd** complex was estimated to be *M*_w_ = 106,000 (corresponding to 130 mer) and *M*_n_ = 18,000, *M*_w_/*M*_n_ = 5.88 compared to polystyrene standards. As an experimental reference data, dynamic light scattering (DLS) measurement of **BPI-2**/**Pd** complex provided an average particle size of 372 nm with a size distribution of 143–2,114 nm in CHCl_3_ (Supplementary Figure 3). These results indicate that **BPI**/**Pd** complexes have polymeric structures.

### Cyclic Voltammetry

**Figure 5:**
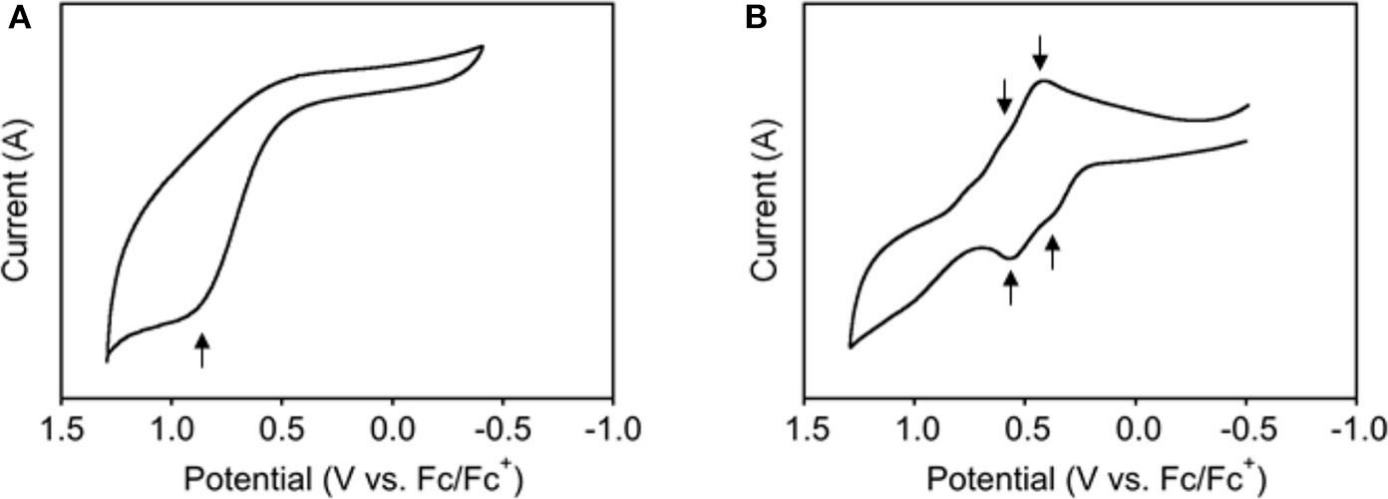
Cyclic voltammograms (V vs. Fc/Fc^+^) of **(A) BPI-2** and **(B) BPI-2/Pd** polymer in CH_2_Cl_2_. Scan rate, 100 mV/s; working electrode, Pt; supporting electrolyte, 0.1 M Bu_4_NClO_4_.

The electrochemical properties, especially for oxidation, of the metal-organic 1D-CP, were studied by cyclic voltammetry (CV) in CH_2_Cl_2_ containing 0.1 M tetrabutylammonium perchlorate (Bu_4_NClO_4_) as supporting electrolyte. The cyclic voltammograms (V vs. Fc/Fc^+^) of **BPI-2** monomer and **BPI-2**/**Pd** polymer are shown in Figure 5. **BPI-2** showed an irreversible oxidation peak at around 0.8 V, where the oxidation may bring about the chemical reaction and/or decomposition of the imine groups. Differential pulse voltammetry (DPV) also provided the corresponding peak at 0.78 V (Supplementary Figure 4A). However, in sharp contrast, **BPI-2**/**Pd** polymer provided reversible multi redox peaks in CV at around *E*_1/2_ = 0.35 and 0.5 V, where the electrochemical properties of imino group may be changed upon complexation with Pd(II) ion. DPV also provided the corresponding two peaks at 0.33 and 0.50 V, respectively (Supplementary Figure 4B). This observed multi-redox behavior of **BPI-2**/**Pd** polymer could be explained by the π-electronic interactions between bipyrrole ligands via Pd(II) ion (Hildebrandt et al., 2011). The positive charge generated upon first oxidation on the polymer backbone withdraw π-electrons of other **BPI** ligands via the metal ions, resulting in a positive shift of their oxidation potential to give the multi redox profile.

### Chirality Induction in BPI-3/Pd Polymer

**BPI** can attach a variety of substituent groups on imino-nitrogens. We have previously synthesized *N*-hexyl **BPI** and found in the crystal structure that two pyrrole components adopt a twisted geometry with an anti-folded confirmation through the phenylene spacer (Ie et al., 2015). The dihedral angle of pyrrole and phenylene components is 33.3° on average, owing to the steric repulsions between the ethyl group attached to the pyrrole-β and phenylene ring (Li et al., 2020). This observed twisted structure allows our expectation that **BPI/Pd** polymer can form a helical CP by attachment of chiral substituent groups on imino-nitrogens (Huang et al., 2018; Wang et al., 2019; Zhou et al., 2020).

We then synthesized **BPI-3(*R*)** and **BPI-3(*S*)**, having chiral alkyl chains on imino-nitrogens. **BPI-3**, upon mixing with Pd(OAc)_2_, also formed the coordination polymer under the same conditions of synthesizing **BPI-2/Pd** polymer (Supplementary Figure 5). However, their average molecular weights [**BPI-3(*R*)/Pd**, *M*_w_ = 10,900, *M*_n_ = 6,800, *M*_w_/*M*_n_ = 1.60; **BPI-3(*S*)/Pd**, *M*_w_ = 8,600, *M*_n_ = 4,700, *M*_w_/*M*_n_ = 1.80] are clearly lower than that of **BPI-2/Pd** polymer (Supplementary Figure 6). The 1-methyl group attached to the *N*-alkyl chain may increase steric crowdness around metal-ligand coordination bonds to decrease the polymerization degree of the CP (Guzei and Wendt, 2006; Talarico and Budzelaar, 2008). Here, **BPI** components may adopt a chiral conformation in the resulting polymer structure.

**Figure 6:**
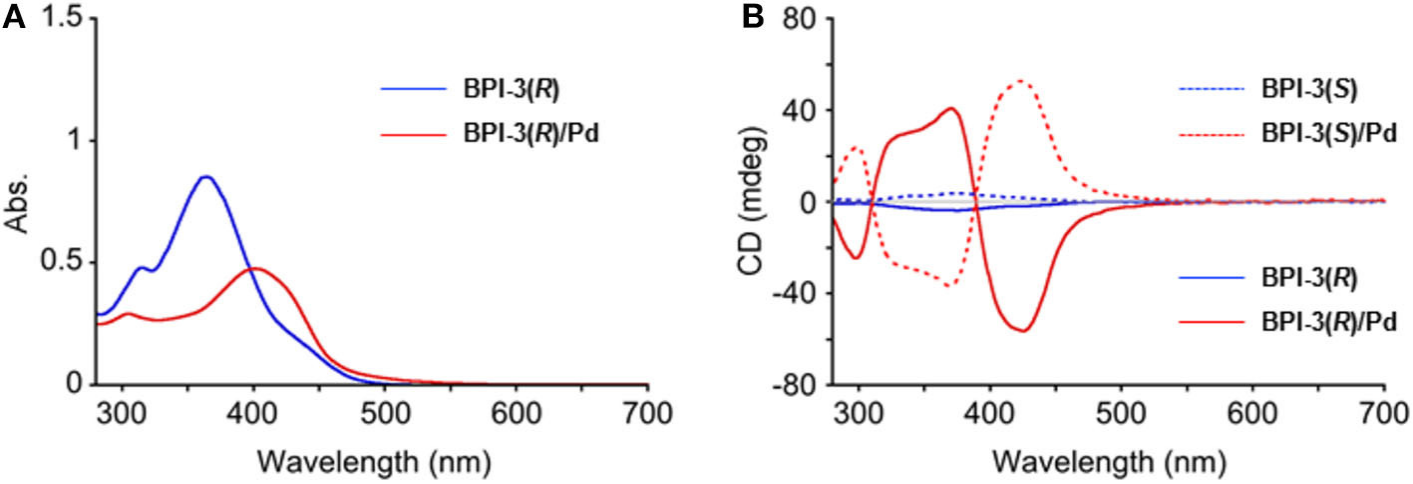
**(A)** UV-Vis absorption spectra of **BPI-3(*R*)** and **BPI-3(*R*)**/**Pd** polymer and **(B)** CD spectra of **BPI-3(*R*)**, **BPI-3(*S*)**, **BPI-3(*R*)**/**Pd** polymer, and **BPI-3(*S*)**/**Pd** polymer in CH_2_Cl_2_ at 25°C. [**BPI-3**] = 3.29 × 10^−5^ M, [**BPI-3**/**Pd** polymer (monomer)] = 3.11 × 10^−5^ M.

**Figure 7:**
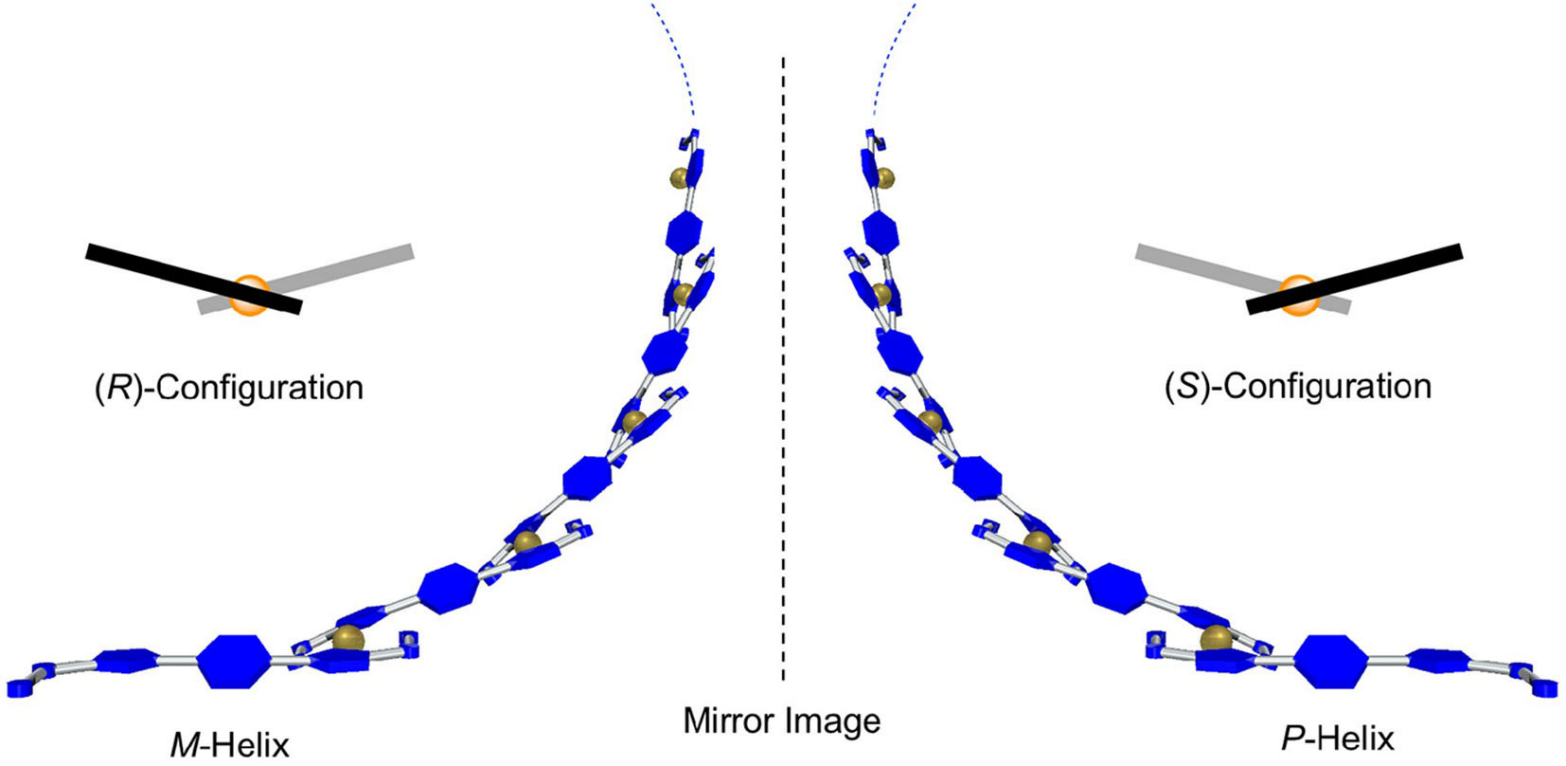
Schematic illustrations of helical polymer structures of **BPI-3(*R*)/Pd (Right)** and **BPI-3(*S*)/Pd (Left)** expected from exciton coupled CD spectra in Figure 6B.

In circular dichroism (CD) spectroscopy, **BPI-3(*R*)** and **BPI-3(*S*)** ligands showed weak CD spectra with λ_max_ at 425 nm (Figure 6). However, interestingly, strong CD responses with Cotton effects appeared in their coordination polymers. **BPI-3(*R*)/Pd** and **BPI-3(*S*)/Pd** showed negative to positive and positive to negative Cotton effects, respectively, at the lowest energy absorption band to give the mirror-imaged CD profiles in each other. These observed spectral features may originate from exciton-coupled CD owing to the helical orientation of **BPI-3** ligands in the 1D-polymeric structure as schematically illustrated in Figure 7. Their Cotton effects allow empirical estimation of their absolute configurations (Gonnella et al., 1982; Furo et al., 2005; Telfer et al., 2011). **BPI-3(*R*)/Pd** and **BPI-3(*S*)/Pd** may dominantly adopt (*S*)- and (*R*)-configuration, which allows the formation of *P*- and *M*-helical coordination polymer, respectively.

Next, we synthesized a coordination copolymer (CCP) with a 1:5 mixture of chiral **BPI-3(*R*)** and achiral **BPI-4** upon mixing with Pd(OAc)_2_ through the similar procedures to the synthesis of the above polymers. The polymer was obtained in 72% yield and characterized using ^1^H NMR, UV-Vis absorption, and CD spectra and SEC. The SEC profile provided only a broad single peak, a typical polydispersion profile of the polymer, indicating random copolymerization. Its average molecular weight is estimated to be *M*_w_ = 11,100 and *M*_n_ = 6,300, *M*_w_/*M*_n_ = 1.76 compared to polystyrene standards in SEC. Hence, its sample solution in CH_2_Cl_2_ was CD active, and the spectral pattern observed was essentially the same as that of **BPI-3(*R*)/Pd** polymer. However, since the spectral intensity is ~4–5 times smaller, it seems that the local chiral twisted conformations occurred in **BPI-3(*R*)/Pd** complexation in the copolymer is not amplified on the resulting polymer backbone. The *p*-phenylene-spacer of **BPI** unit, which provides a distance between its substituent groups, likely allows the polymerization without steric repulsions and/or interactions of the chiral metal-coordination moieties.

## Conclusion

In this study, we have synthesized a novel metal-organic 1D-coordination polymer composed of a phenylene-bridged bipyrrole bearing *N*-alkylimino groups and palladium(II) ion. Although most of 1D-CPs reported are unstable in solutions, BPI and Pd(II) ion form a metal coordinated π-conjugation polymer, which is stable in the solutions. BPI/Pd polymer showed π-electronic communications between the BPI components via Pd(II) ion in its polymer structure to give exciton coupling in the absorption spectrum. Further it allowed a reversible multi redox in cyclic voltammetry. BPI-3/Pd polymer having chiral alkyl chains on the imino-nitrogen showed strong circular dichroism (CD) owing to its one-handedly twisted helical polymer structure. We can expect a variety of the molecular functions through structural modifications of *N*-substituent groups, metal ion, and aromatic spacer, and control of the polymerization degree of this metal-organic 1D-coordination polymer.

## Data Availability Statement

The raw data supporting the conclusions of this article will be made available by the authors, without undue reservation.

## Author Contributions

All authors listed have made a substantial, direct and intellectual contribution to the work, and approved it for publication.

## Conflict of Interest

The authors declare that the research was conducted in the absence of any commercial or financial relationships that could be construed as a potential conflict of interest.
