# Supplementary material for: A Chiral Metal-Organic 1D-Coordination Polymer Upon Complexation of Phenylene-Bridged Bipyrrole and Palladium (II) Ion
Source: Front Chem. 2020 Dec 1;8:613932. doi: 10.3389/fchem.2020.613932 (PMC7736045; doi:10.3389/fchem.2020.613932)
Supplement: Supplementary file 1 [file Data_Sheet_1.PDF]

Supporting Information

A Chiral Metal-Organic 1D-Coordination Polymer upon Complexation of Phenylene-bridged Bipyrrole and Palladium (II) Ion

Kumiko Nishinaka[2], Jiandong Han[1], Dongli Han[1], Yue Liu[2], Yanqing Du[1], Meiling Wang[1], Chaolu Eerdun[1]*, Nobuyasu Naruse[3], Yutaka Mera[3], Yoshio Furusho[3] and Akihiko Tsuda[1,2,3]*

[1] *Department of Pharmaceutical Sciences, Inner Mongolia Medical University, Jinshan Economic and Technology Development District, Hohhot, Inner Mongolia 010110, China*

[2] *Department of Chemistry, Graduate School of Science, Kobe University, 1-1 Rokkodai-cho, Nada-ku, Kobe 657-8501, Japan*

[3] *Department of Chemistry, Shiga University of Medical Science, Seta Tsukinowa-cho, Otsu, Shiga 520-2192, Japan*

1. Materials S1

2. Measurements S1

3. Synthesis S2

4. ^{1}H and ^{13}C NMR spectra S6

5. ^{1}H NMR spectra of **BPI-2** and **BPI-2/Pd** complex S11

6. UV-Vis absorption spectra of **BPI-2** and **BPI-2/Pd** complex S12

7. DLS of **BPI-2/Pd** complex S12

8. CV of **BPI-2** and **BPI-2/Pd** complex S13

9. ^{1}H NMR spectra of **BPI-3(*R*)** and **BPI-3(*R*)/Pd** complex S13

10. SEC of **BPI-3(*R*)/Pd** complex S14

11. SEC of **BPI-3(*R*)/BPI-4/Pd** complex S14

12. References S15

1. Materials

Unless otherwise noted, reagents and solvents were used as received from Tokyo Chemical Industry Co., Ltd. [benzylamine (>99.5%), aminododecane (>99.5%)], Sigma-Aldrich, Inc. [(*R*)-2-aminononane (>99.5%), (*S*)-2-aminononane (>99.5%)], InnoChem, Inc. [TFA (>99.5%), CH_3OH (>99.0%), CH_2Cl_2 (>99.0%), Na_2SO_4 (>99.0%), K_2CO_3 (>99.0%), palladium(II) acetate (>99.5%)].

2. Measurements

Measurements were taken at 293 K unless otherwise indicated. ^1H and ^{13}C NMR spectra were recorded on Bruker AVANCE 400/600 spectrometers, where chemical shifts (δ in ppm) were determined with respect to tetramethylsilane as the internal standard. Absorption spectra were recorded on a JASCO V-670 UV/VIS/NIR spectrometer equipped with a JASCO ETC-717 temperature/stirring controller. Fluorescence spectra were recorded on a JASCO ETC-273T spectrometer equipped with a JASCO FP-6500 temperature/stirring controller. CD spectra were recorded on a JASCO J-820F. HRMS were recorded on a LTQ Orbitrap Discovery (Thermo Fisher Scientific) with CH_3OH as a solvent.

Analytical HPLC was performed at 313 K using THF as an eluent through TOSOH TSKgel G4000H_{HR} and G5000H_{HR} columns on a JASCO Type PU-2089 quaternary gradient pump, equipped with a JASCO Type MD-2018 photodiode array detector and a CO4060 intelligent column thermostat. Polystyrene standards with molecular weight 906, 1300, 2000, 4000, 13502, 25000, 50000, and 123000 g/mol were used in the calibration of the molecular weights of the polymers.

Electrochemical measurements were performed with an ALS 1210A electrochemical analyzer using a glassy carbon working electrode, a platinum wire counter electrode, and an Ag/Ag^+ [0.01 M $AgNO_3$, 0.1 M Bu_4NClO_4 (MeCN)] reference electrode. The scan rate was 500–50 $mV\cdot s^{-1}$. The CV curves were calibrated using the ferrocene/ferrocenium

(Fc/Fc^+) redox couple as an external standard, which was measured under the same conditions before and after the measurement of samples.

3. Synthesis

Synthetic Scheme

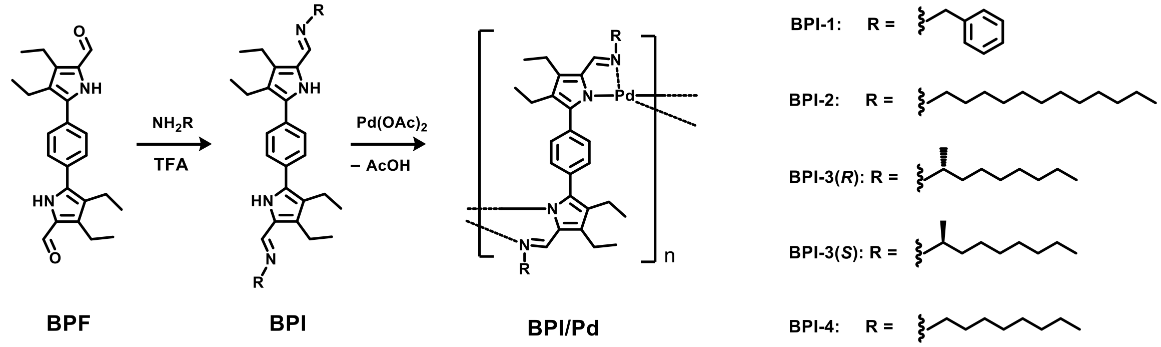

Synthetic procedures

1,4-Bis(3,4-diethyl-5-formylpyrr-2-yl)benzene **BPF** was prepared by the procedures analogous to that reported previously, and unambiguously characterized by means of ^1H NMR spectroscopy.[1-3] **BPI**, **BPI/Pd** was prepared through modifications of the literature methods.[4]

1,4-Bis(5-benzylimino-3,4-diethylpyrr-2-yl)benzene (BPI-1):

1,4-Bis(3,4-diethyl-5-formylpyrr-2-yl)benzene **BPF** (100 mg, 0.26 mmol) was mixed with benzylamine (0.6 mL, 5.5 mmol) in a CH_3OH solution (10 mL) containing TFA (0.2 mL) and CH_2Cl_2 (0.2 mL). The sample solution was stirred at room temperature overnight to give a brown precipitate. It was then filtered to leave **BPI-1** as a blown solid in 96 % yield. ^1H NMR (400 MHz, $CDCl_3$, 293 K): δ 8.27 (s, 2H, CH=N), 7.49 (s, 4H, phenylene-H), 7.35−7.23 (m, 10H, phenyl-H), 4.73 (s, 4H, benzyl-H), 2.67 (q, 4H, J = 7.5 Hz, CH_2), 2.63 (q, 4H, J = 7.5 Hz, CH_2) 1.24 (t, 6H, J = 7.5 Hz, CH_3), 1.21 (t, 6H, J = 7.5 Hz, CH_3); HRMS: m/z calculated for $[M+H]^+$ ($C_{38}H_{42}N_4$) 555.3443, found 555.3480.

1,4-Bis(5-*n*-dodecylimino-3,4-diethylpyrr-2-yl)benzene (BPI-2):

1,4-Bis(3,4-diethyl-5-formylpyrr-2-yl)benzene **BPF** (500 mg, 1.33 mmol) was mixed with 1-aminododecane (493.1 mg, 2.60 mmol) in a H_2O solution (10.5 mL) containing CH_2Cl_2 (13.8 mL). The sample solution was stirred at room temperature overnight. It was then that was washed with water, and the organic layer was extracted. The sample solution was dried over Na_2SO_4, and evaporated to leave a brown solid as the product in 75% yield. ^{1}H NMR (400 MHz, $CDCl_3$, 293 K) : δ 8.12 (s, 2H, CH=N), 7.51 (s, 4H, phenylene-H), 3.52 (t, 4H, J = 7.0 Hz, N-CH_2), 2.64 (q, 8H, J = 7.5 Hz, CH_2), 1.64 (m, 20H, J = 7.5 Hz, dodecyl-H), 1.32−1.19 (m, 12H, J = 7.5 Hz, CH_3), 0.88 (t, 6H, J = 7.5 Hz, CH_3) ; ^{13}C NMR (100 MHz, $CDCl_3$, 293 K) : δ 149.39, 131.36, 131.05, 130.79, 127.00, 125.91, 123.24, 61.42, 31.93, 31.41, 29.68, 29.66, 29.51, 29.37, 27.33, 22.70, 17.60, 17.50, 17.04, 16.18, 14.14; HRMS: m/z calculated for $[M+H]^+$ ($C_{48}H_{78}N_4$) 711.6260, found 711.6291.

(1*E*,1'*E*)-1,1'-(1,4-phenylenebis(3,4-diethyl-1H-pyrrole-5,2-diyl))bis(*N*-((*R*)-nonan-2-yl)methanimine) (BPI-3(*R*)):

1,4-Bis(3,4-diethyl-5-formylpyrr-2-yl)benzene **BPF** (19.4 mg, 0.05 mmol) was mixed with (*R*)-2-aminononane (14.8 mg, 0.1 mmol) in a THF solution (0.3 mL). The sample solution was stirred at room temperature overnight to give a brown precipitate. It was then filtered to give the product in 86 % yield. ^{1}H NMR (600 MHz, $CDCl_3$, 293 K) : δ 8.12 (s, 2H, CH=N), 7.51 (s, 4H, phenylene-H), 3.20 (s, 2H, J = 7.5 Hz, N-CH), 2.64 (q, 8H, J = 7.5 Hz, CH_2), 1.39−1.31(m, 24H, J = 7.5 Hz, heptyl-H), 1.35−1.25(m, 12H, J = 7.5 Hz, CH_3), 0.85 (t, 6H, J = 6.5 Hz, CH_3), 0.72 (t, 6H, J = 7.5 Hz, CH_3); ^{13}C NMR (151 MHz, $CDCl_3$, 293 K) : δ 147.41, 131.42, 130.76, 127.31, 127.12, 125.84, 123.31, 107.75, 77.23, 77.02, 76.81, 67.42, 66.42, 38.19, 31.89, 31.85, 31.78, 29.56, 29.49, 29.32, 29.23, 29.03, 26.65, 26.42, 23.00, 22.67, 22.58, 17.60, 17.48, 17.41, 17.32, 17.05, 16.13, 15.90, 14.10, 14.04. HRMS: m/z calculated for $[M+H]^+$ ($C_{42}H_{66}N_4$) 627.5321, found 627.5340.

(1*E*,1'*E*)-1,1'-(1,4-phenylenebis(3,4-diethyl-1H-pyrrole-5,2-diyl))bis(*N*-((*S*)-nonan-2-yl)methanimine) (BPI-3(*S*)):

1,4-Bis(3,4-diethyl-5-formylpyrr-2-yl)benzene **BPF** (23.5 mg, 0.06 mmol) was mixed with (*S*)-2-aminononane (17.06 mg, 0.1 mmol) in a THF solution (0.3 mL). The sample solution was stirred at room temperature overnight to give a brown precipitate. It was then filtered to give the product in 92 % yield. ^{1}H NMR (600 MHz, $CDCl_3$, 293 K) : δ 8.12 (s, 2H, CH=N), 7.51 (s, 4H, phenylene-H), 3.20 (s, 2H, J = 7.5 Hz, N-CH), 2.64 (q, 8H, J = 7.5 Hz, CH_2), 1.39−1.31(m, 24H, J = 7.5 Hz, heptyl-H), 1.35−1.25(m, 12H, J = 7.5 Hz, CH_3), 0.85 (t, 6H, J = 6.5 Hz, CH_3), 0.72 (t, 6H, J = 7.5 Hz, CH_3); ^{13}C NMR (151 MHz, $CDCl_3$, 293 K) : δ 147.41, 131.42, 130.76, 127.31, 127.12, 125.84, 123.31, 107.75, 77.23, 77.02, 76.81, 67.42, 66.42, 38.19, 31.89, 31.85, 31.78, 29.56, 29.49, 29.32, 29.23, 29.03, 26.65, 26.42, 23.00, 22.67, 22.58, 17.60, 17.48, 17.41, 17.32, 17.05, 16.13, 15.90, 14.10, 14.04. HRMS: m/z calculated for $[M+H]^+$ ($C_{42}H_{66}N_4$) 627.5321, found 627.5340.

BPI-1/Pd

BPI-1 (30.5 mg, 0.055 mmol) was added to a CH_2Cl_2 solution (5 mL) containing $Pd(OAc)_2$ (24.6 mg, 0.11 mmol). The sample solution was stirred at room temperature for 54 h, and then washed with aqueous K_2CO_3. It was extracted with CH_2Cl_2, where the combined organic extract was dried over Na_2SO_4, and the solvent was removed by evaporation. The residue was washed with CH_3OH, and filtered to leave **BPI-1/Pd** complex as orange solid (32 mg, 76 % yield).

BPI-2/Pd

BPI-2 (39.1mg, 0.055mmol) was added to a CH_2Cl_2 solution (2.8 mL) containing $Pd(OAc)_2$ (24.6 mg, 0.11 mmol). The sample solution was stirred at room temperature for 54 h, and then washed with aqueous K_2CO_3. It was extracted with CH_2Cl_2, where the combined organic extract was dried over Na_2SO_4, and the solvent was removed by

evaporation. The residue was washed with CH_3OH, and filtered to leave **BPI-2/Pd** complex as reddish orange solid (28 mg, 56 % yield).

BPI-3(*R*)/Pd

BPI-3(*R*) (32.6 mg, 0.052 mmol) was added to a CH_2Cl_2 solution (2.7 mL) containing $Pd(OAc)_2$ (23.2 mg, 0.10 mmol). The sample solution was stirred at room temperature for 22 h, and then washed aqueous K_2CO_3. It was extracted with CH_2Cl_2, where the combined organic extract was dried over Na_2SO_4, and the solvent was removed by evaporation. The residue was washed with CH_3OH, and filtered to leave **BPI-3(*R*)/Pd** as brown solid (23 mg, 65 % yield).

BPI-3(*S*)/Pd

BPI-3(*S*) (27.5 mg, 0.044 mmol) was added to a CH_2Cl_2 solution (2.3 mL) containing $Pd(OAc)_2$ (19.6 mg, 0.09 mmol). The sample solution was stirred at room temperature for 22 h, and then washed with aqueous K_2CO_3. It was extracted with CH_2Cl_2, where the combined organic extract was dried over Na_2SO_4, and the solvent was removed by evaporation. The residue was washed with CH_3OH, and filtered to leave **BPI-3(*S*)/Pd** as brown solid (24 mg, 79 % yield).

4. ^{1}H and ^{13}C NMR spectra

BPI-1

^{1}H NMR (400 MHz, $CDCl_3$, 293 K)

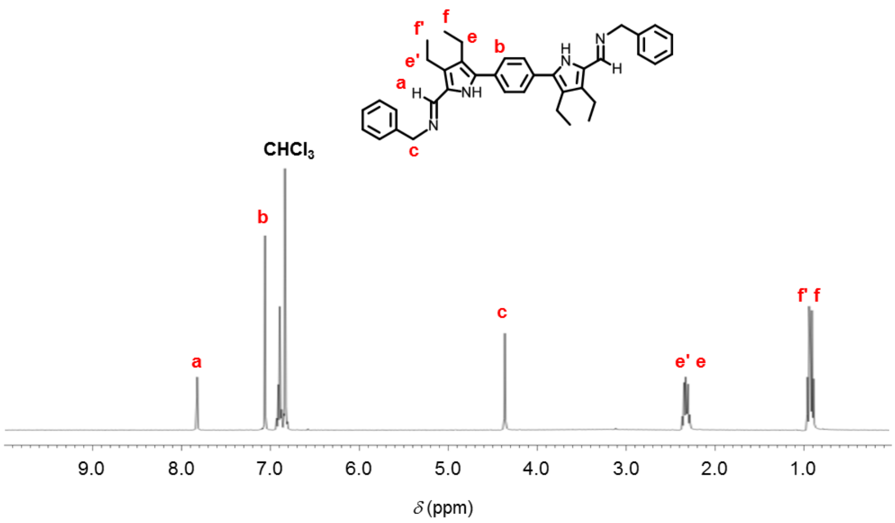

BPI-2

^{1}H NMR (400 MHz, $CDCl_3$, 293 K)

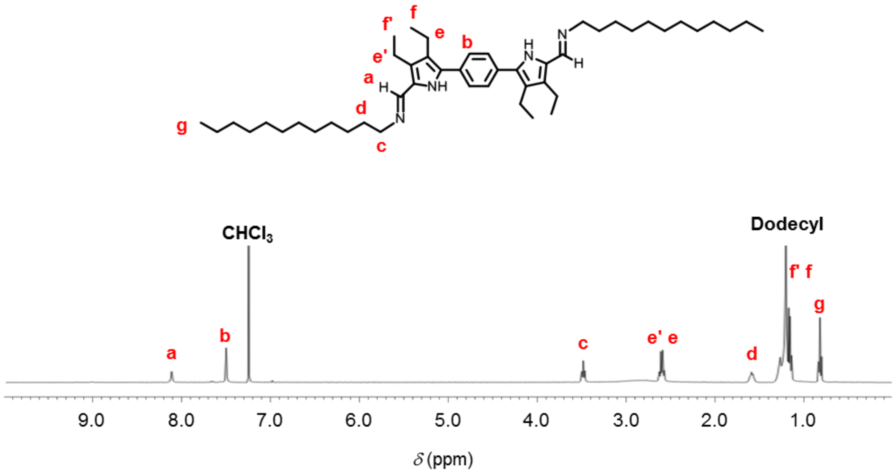

BPI-3(*R*)

^{1}H NMR (600 MHz, $CDCl_3$, 293 K)

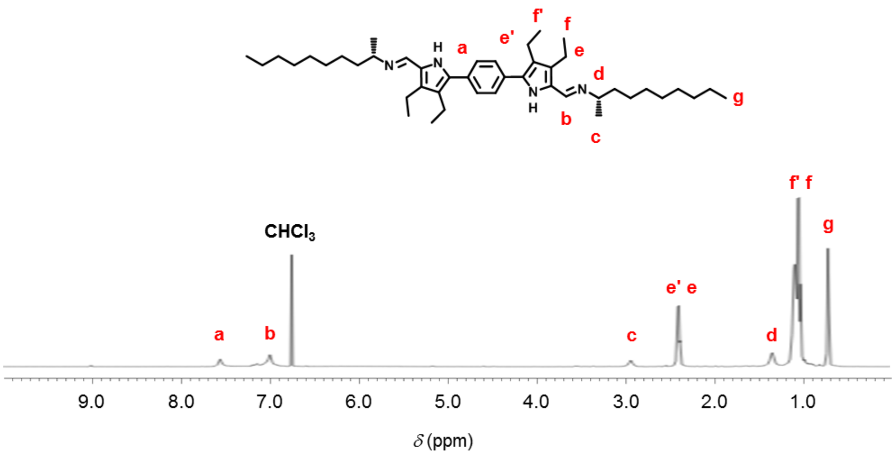

^{13}C NMR (151 MHz, $CDCl_3$, 293 K)

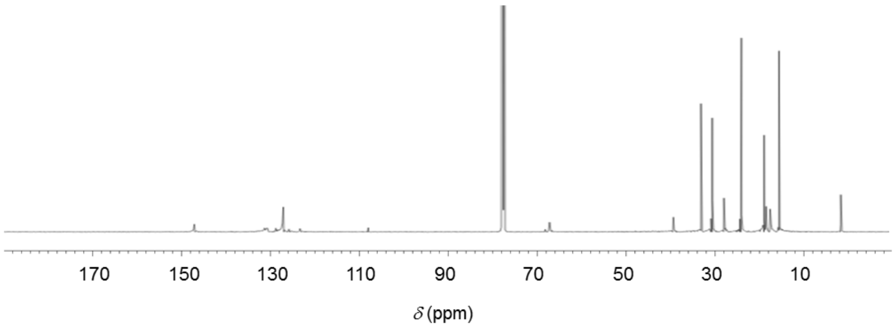

BPI-3(*S*)

^{1}H NMR (600 MHz, $CDCl_3$, 293 K)

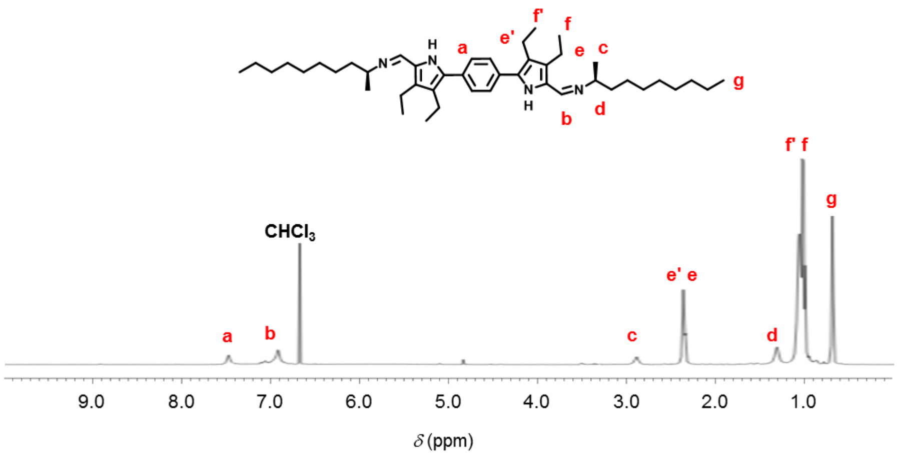

^{13}C NMR (151 MHz, $CDCl_3$, 293 K)

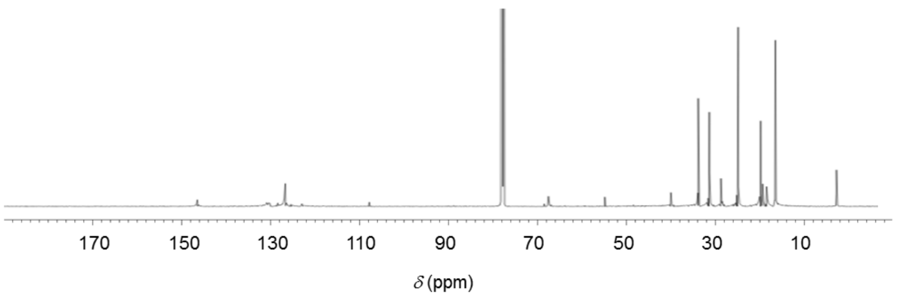

BPI-1/Pd

^{1}H NMR (400 MHz, $CDCl_3$, 293 K)

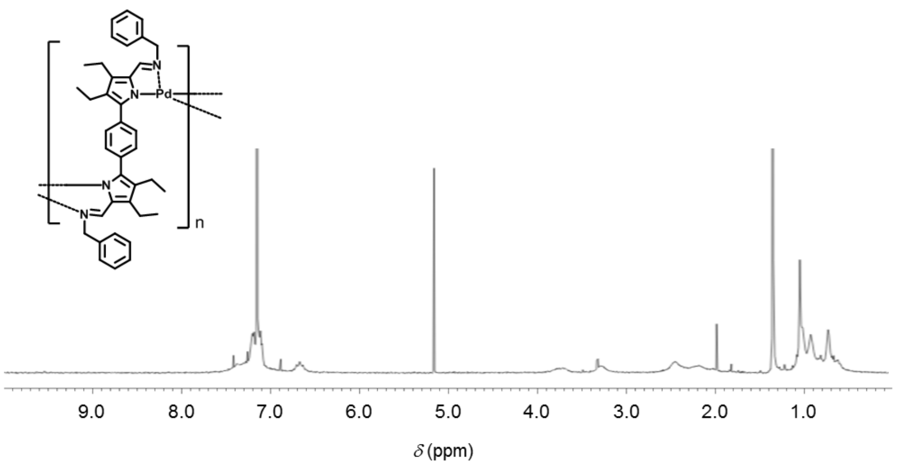

BPI-2/Pd

^{1}H NMR (400 MHz, $CDCl_3$, 293 K)

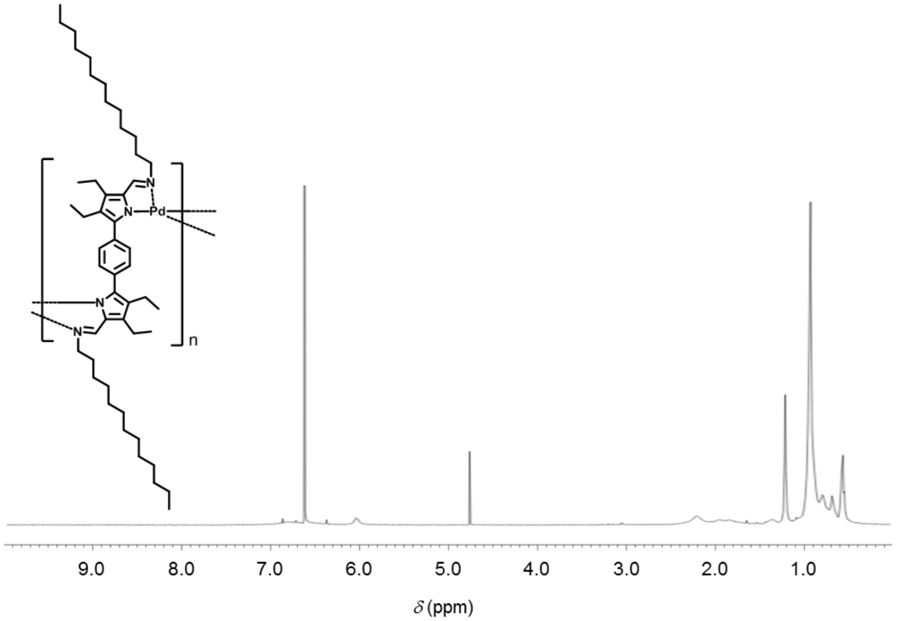

BPI-3(*R*)/Pd

^{1}H NMR (600 MHz, $CDCl_3$, 293 K)

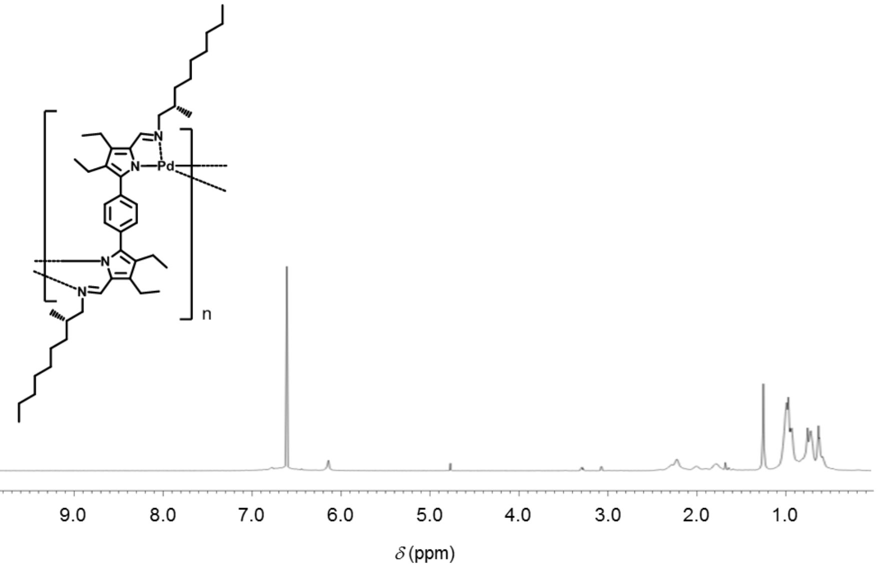

BPI-3(*S*)/Pd

^{1}H NMR (600 MHz, $CDCl_3$, 293 K)

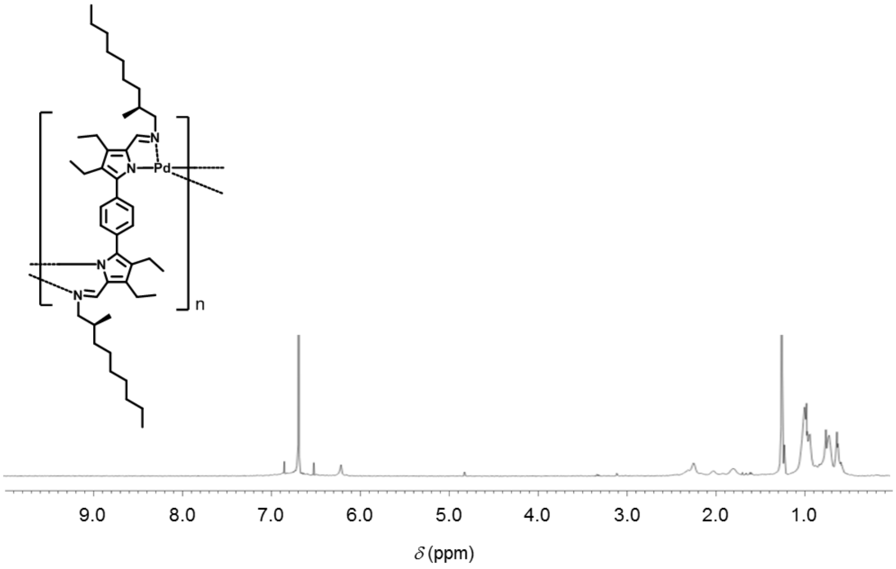

5. ^{1}H NMR spectra of BPI-2 and BPI-2/Pd complex

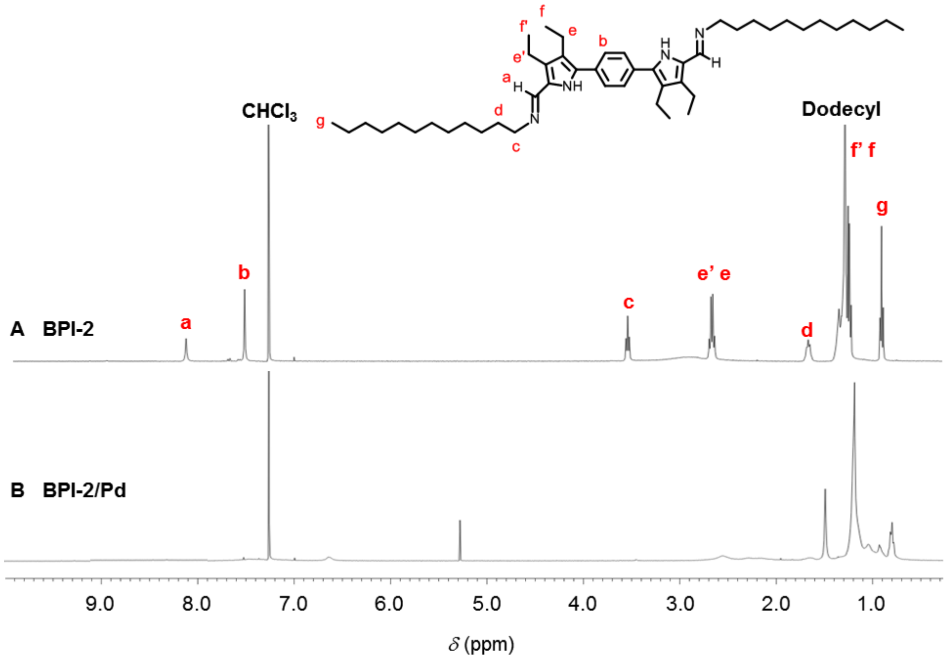

Figure S1. ^{1}H NMR spectra (400 MHz, $CDCl_3$) of (A) **BPI-2** and (B) a product formed upon mixing **BPI-2** and $Pd(OAc)_2$ in 1:1 ratio.

6. UV-Vis absorption spectra of BPI-2 and BPI-2/Pd complex

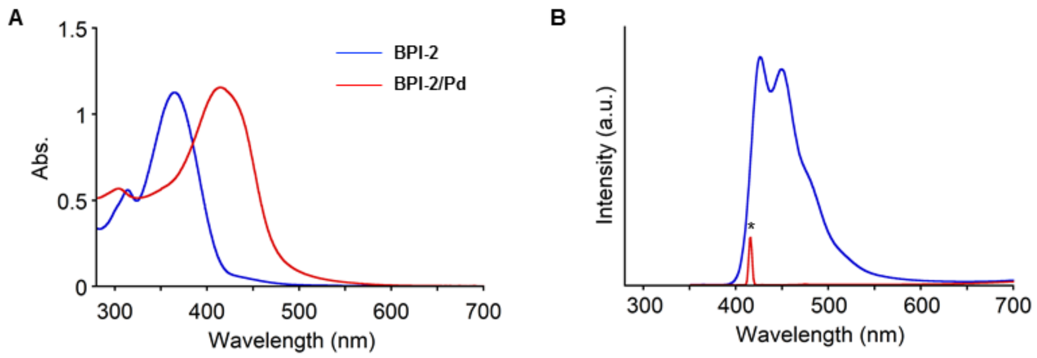

Figure S2. (A) UV-Vis absorption spectra and (B) fluorescence spectra upon excitation at 364.5 nm and 413.5 nm of **BPI-2** and **BPI-2**/**Pd** at 298 K. [**BPI-2**] = 2.48 × 10^{-5} M, [**BPI-2**/**Pd** complex] = 2.06 × 10^{-5} M. *Stray light.

7. DLS of BPI-2/Pd complex

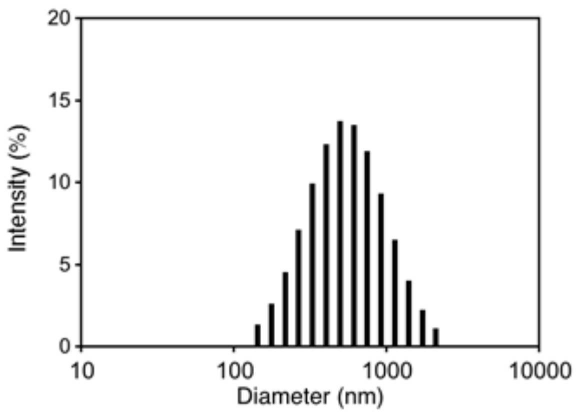

Figure S3. DLS histogram profile of **BPI-2**/**Pd** complex in $CHCl_3$.

8. CV of BPI-2 and BPI-2/Pd complex

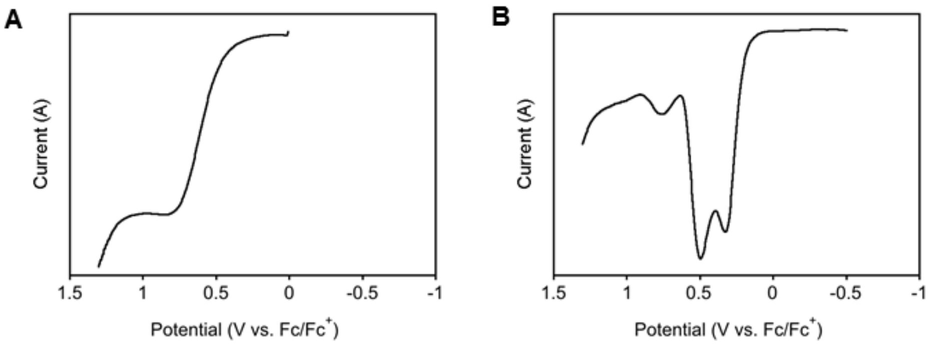

Figure S4. Differential pulse voltammograms (V vs. Fc/Fc^{+}) of (A) **BPI-2** and (B) **BPI-2/Pd** complex in CH_2Cl_2. Scan rate, 100 mV/s; working electrode, Pt; supporting electrolyte, 0.1 M Bu_4NClO_4.

9. ^{1}H NMR spectra of BPI-3(*R*) and BPI-3(*R*)/Pd complex

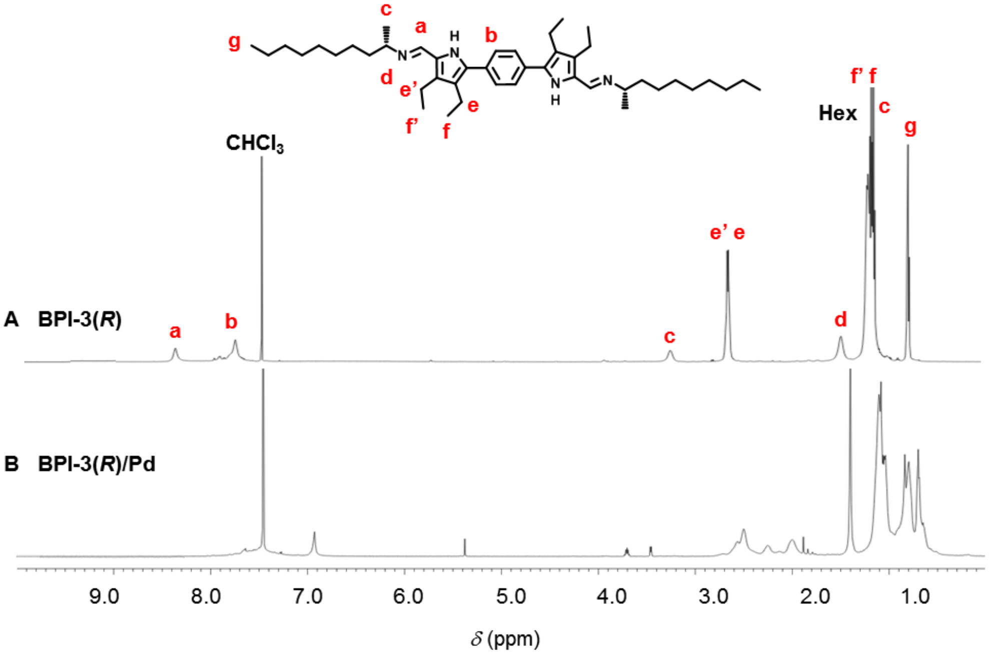

Figure S5. ^{1}H NMR spectra (600 MHz, $CDCl_3$) of (A) **BPI-3(*R*)** and (B) a product formed upon mixing **BPI-3(*R*)** and $Pd(OAc)_2$ in 1:1 ratio.

10. SEC of BPI-3(*R*)/Pd complex

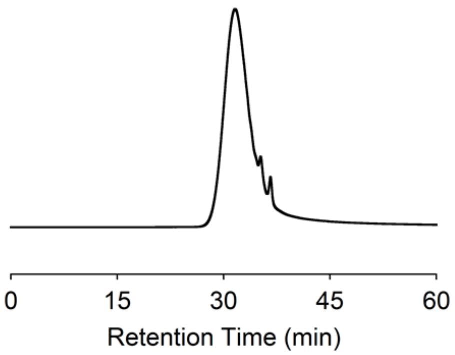

Figure S6. SEC profile of **BPI-3(*R*)/Pd** complex monitored at 420 nm in SEC. [Conditions] Column: TOSOH TSKgel G4000H_{HR} and G5000H_{HR}, Solvent: THF, Flow speed: 0.5 mL/min, Temperature: 293 K

11. SEC of BPI-3(*R*)/BPI-4/Pd complex

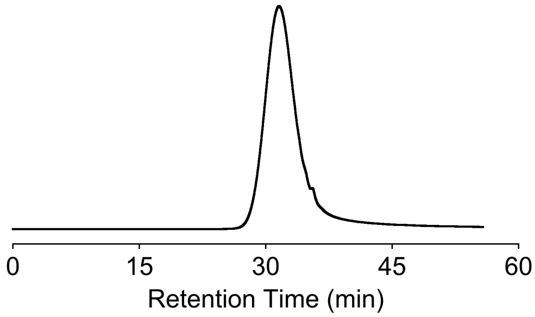

Figure S7. SEC profile of a polymer formed upon mixing **BPI-3(*R*)**, **BPI-4**, and $Pd(OAc)_2$ in 1:5:6 ratio monitored at 418 nm in SEC. [Conditions] Columns: TOSOH TSKgel G4000H_{HR} and G5000H_{HR}, Solvent: THF, Flow speed: 0.5 mL/min, Temperature: 293 K

12. References

1. Setsune, J. I., Toda, M., Yoshida, T., Imamura, K., and Watanabe, K. The synthesis and dynamic structures of multinuclear complexes of large porphyrinoids expanded by phenylene and thienylene spacers. *Chemistry–A European Journal*, **2015**, *21*, 12715-12727
2. A. Tsuda, K. Imamura, *JP Pat., 2018-118944,* **2018**.
3. Imamura, K., Ueno, Y., Akimoto, S., Eda, K., Du, Y., Eerdun, C., et al. An Acid-Responsive Single Trichromatic Luminescent Dye That Provides Pure White-Light Emission. *ChemPhotoChem*, **2017**, *1*, 427.
